# A Phase II Study of Toripalimab in Combination with Gemcitabine and 5-Fluorouracil as First-Line Therapy for Advanced or Metastatic Biliary Tract Carcinoma

**DOI:** 10.3390/cancers18010088

**Published:** 2025-12-27

**Authors:** Fangyong Lei, Wenjing Deng, Ying Zhou, Lilan Fang, Xiuxin Lin, Lingyu Qin, Chunming Li, Jian Rao, Gengsheng Yu

**Affiliations:** Department of Oncology, Jiangmen Central Hospital, Jiangmen 529000, China; wjdeng2011@163.com (W.D.); zhou21341215@126.com (Y.Z.); fangli416@163.com (L.F.); 13822348180@163.com (X.L.); qinlingyu007@163.com (L.Q.); sumsming@163.com (C.L.); 13500288552@163.com (J.R.)

**Keywords:** biliary tract carcinoma, toripalimab, gemcitabine, 5-Fu, PD-L1, tumor mutational burden (TMB)

## Abstract

Background/Objectives: The anti-PD-L1 antibody has been applied for use in first-line advanced biliary duct cancer patients. However, clinical evidence of toripalimab in combination with biweekly 5-fluorouracil (5-FU) is limited and predictive biomarkers of treatment benefits remain unclear. Methods: This prospective study enrolled patients with unresectable or metastatic BTC who received toripalimab in combination with gemcitabine and biweekly 5-FU. The primary endpoints were progression-free survival (PFS) and objective response rate (ORR). Secondary endpoints included overall survival (OS) and safety. Exploratory analyses evaluated associations between programmed cell death-ligand 1 (PD-L1) expression or tumor mutational burden (TMB) and clinical outcomes. Results: A total of 30 patients were enrolled, with a median follow-up duration of 16.0 months. The ORR was 13.0%. The median PFS and OS were 5.3 months (95% CI: 3.59–7.01) and 11.7 months (95% CI: 6.07–17.33), respectively. Subgroup analyses revealed no significant association between PD-L1 status or TMB level and improved PFS. All patients experienced adverse events (AEs), while grade 3–4 AEs occurred in eight patients (26.7%), most commonly anemia (20.0%), leukocytopenia (13.3%), and nausea (6.6%). No grade 5 AEs were observed, and the safety profile was considered manageable. Conclusions: Toripalimab in combination with gemcitabine and 5-fluorouracil showed promising efficacy and was well-tolerated as a first-line therapy in advanced biliary duct cancer patients. Although PD-L1 expression and TMB did not predict treatment benefit, larger studies are needed to validate potential biomarkers and further optimize immunochemotherapy strategies for BTC.

## Simple Summary

Biliary tract carcinoma (BTC) is a rare, aggressive cancer arising from biliary epithelial cells, often diagnosed at advanced stages with a median survival of less than one year. Prior investigations have explored the efficacy of programmed cell death protein 1 (PD-1) inhibitors and gemcitabine-based chemotherapy regimens in advanced BTC. In this phase II study, we evaluated a new regimen for advanced or metastatic BTC using toripalimab combined with gemcitabine and 5-fluorouracil (5-FU). The regimen achieved an objective response rate (ORR) of 13%. Subgroup analyses revealed no significant improvement in outcomes among patients with elevated programmed death-ligand 1 (PD-L1) expression or high tumor mutational burden (TMB). Treatment-related adverse events (TRAEs) occurred in all patients, with grade 3 or higher TRAEs reported in eight patients, including anemia (20%), neutropenia (13.3%), and nausea (6.6%); no treatment-related deaths were observed. Overall, this combination therapy offers a viable alternative for patients with advanced BTC and demonstrates an acceptable safety profile.

## 1. Introduction

Biliary tract carcinoma (BTC) is the second most common primary hepatic malignancy, with a high incidence in Southeast Asia. In China, its prevalence is approximately seven cases per million population [1,2]. Recent epidemiological data show a continuous rise in both incidence and mortality, with nearly 75% of patients presenting with metastatic or locally advanced disease at initial diagnosis. BTC is an adenocarcinoma originating from the biliary epithelium and is classified by tumor location into intrahepatic BTC (ICC), extrahepatic BTC (ECC; including hilar and distal BTC), and gallbladder carcinoma (GBC).

The Advanced Biliary Tract Cancer (ABC)-02 study established the traditional chemotherapy regimen of gemcitabine and cisplatin as the first-line treatment for advanced or metastatic BTC, median progression-free survival (PFS) and overall survival (OS) were 8.0 months and 11.7 months, respectively [3]. Alberto Serrano et al. reported that gemcitabine monotherapy, with varying dosing schedules, achieved an objective response rate (ORR) of 0–36%, while gemcitabine-based combinations with 5-fluorouracil/leucovorin or oxaliplatin yielded a slightly higher ORR of 12–64%, with a median ORR of approximately 30% [4]. For older patients or those ineligible for intensive cisplatin-based chemotherapy, these regimens offer comparable efficacy with reduced toxicity, making them suitable for subgroups with poor performance status or high tumor burden. Research has demonstrated that biweekly gemcitabine combined with a high dose 24 h continuous infusion of 5-FU achieved an objective response rate (ORR) of 21.4% and a disease control rate (DCR) of 67.9% in patients with locally advanced or metastatic BTC [5].

Immune checkpoint expression is increasingly recognized in BTC biology. PD-L1 expression has been detected in 30.5% (54/177) of tumor cells and 43.5% (77/177) of stromal cells in BTC cases [6]. Nakamura et al. similarly demonstrated immune checkpoint molecule upregulation in approximately 45% of BTC samples, with PD-L1 positivity ranging between 30 and 42% [7,8]. Advances in next-generation sequencing has shed light on the genomic landscape of BTC, including alteration in isocitrate dehydrogenase (IDH1/2) mutations and fibroblast growth factor receptor (FGFR) fusions, which offer actionable targets in the subset of BTC patients [9]. However, only a minority of BTC patients harbor such actionable genetic drivers, limiting the overall impact of targeted therapy.

Immunotherapy has recently reshaped the treatment paradigm for advanced BTC. The KEYNOTE-028 and KEYNOTE-158 trials demonstrated durable antitumor activity of pembrolizumab in previously treated, locally advanced, or metastatic BTC, with ORRs of 13.0% and 5.8%, respectively [10]. The global phase III TOPAZ-1 trial demonstrated that durvalumab with gemcitabine plus cisplatin significantly improved OS in patients with advanced BTC, with a hazard ratio (HR) of 0.74 (95% CI, 0.63–0.87) at three years (median OS: 12.9 months vs. 11.3 months) [11]. Similarly, the KEYNOTE-966 trial showed that pembrolizumab combined with gemcitabine plus cisplatin extended OS, with an HR of 0.83 (95% CI, 0.72–0.95), reducing the risk of death by 17% (median OS: 12.7 months vs. 10.9 months) [12].

Toripalimab is a selective, recombinant humanized IgG4 monoclonal antibody targeting PD-1, blocking its interaction with PD-L1 and PD-L2 ligands and thereby preventing T-cell exhaustion while enhancing antitumor immunity [12,13]. Toripalimab has demonstrated efficacy across multiple tumor types in clinical trials. Building on these findings, the present study aims to evaluate the efficacy and safety of toripalimab combined with gemcitabine and 5-FU as a first-line treatment for patients with advanced or metastatic BTC.

## 2. Materials and Methods

### 2.1. Study Design

This phase II clinical trial evaluates the efficacy of toripalimab combined with biweekly gemcitabine and 5-fluorouracil (5-FU) in patients with advanced BTC, with a total enrollment of 30 patients.

### 2.2. Eligibility

The primary eligibility criteria included histologically confirmed, unresectable, locally advanced or metastatic BTC, including intrahepatic and extrahepatic cholangiocarcinoma or gallbladder carcinoma. Key inclusion criteria for patients were as follows: aged between 18 and 70 years old; life expectancy of more than 3 months; adequate hematological, hepatic, and renal function; appropriately managed biliary tract obstruction before study entry with total bilirubin concentration ≤ 2 × ULN (upper limit number); at least one measurable metastatic disease based on Response Evaluation Criteria in Solid Tumors (RECIST) version 1.1; availability of sufficient biopsy specimens for whole-exome sequencing and PD-L1 assessment; no prior systemic therapy for advanced or metastatic disease, with completion of adjuvant chemotherapy more than six months before enrollment using regimens that excluded gemcitabine or 5-FU. All patients provided written informed consent. Exclusion criteria included the following: concurrent malignancy other than BTC; central nervous system metastasis; unresolved biliary obstruction; active infections requiring treatment; noninfectious pneumonitis requiring corticosteroid therapy; active autoimmune diseases; ongoing systemic immunosuppressive treatment.

### 2.3. Treatment and Endpoints

Following enrollment, patients received toripalimab at 3 mg/kg intravenously on days 1 and 15, in combined with gemcitabine plus 5-FU. The chemotherapy regimen consisted of gemcitabine 1250 mg/m^2^, leucovorin 200 mg/m^2^, 5-FU 400 mg/m^2^ bolus, followed by 48 h continuous infusion of 5-FU at 2.4 g/m^2^ on days 1 and 15. Each treatment cycle lasted 4 weeks, and a total of four cycles were administered. After completion of combination therapy, patients received maintenance therapy with toripalimab 240 mg every 3 weeks, continuing every 3 weeks until disease progression, unacceptable toxicity, withdrawal of consent, or a total treatment period of 1 year. Decisions to continue therapy beyond radiographic progression were made jointly by investigators and patients. Upon disease progression, patients were offered alternative systemic therapies, including mFOLFOX6 (5-FU, leucovorin, and oxaliplatin), gemcitabine-oxaliplatin (Gemox), and Lenvatinib [14]. HER2-positive patients were eligible for targeted therapies with trastuzumab and(or) pertuzumab [15,16]. Patients with FGFR alterations were eligible for FGFR inhibitors when applicable [17]. Patients with R0 potential resection margins underwent surgical intervention. Additional local therapies, including radiofrequency ablation, transarterial chemoembolization (TACE), and palliative radiotherapy, were considered when applicable [18,19,20]. Treatment was discontinued upon investigator’s assessment of disease progression, intolerable toxicity, or patient withdrawal of consent. This study was conducted in accordance with the Declaration of Helsinki (revised in 2013) and was approved by the institutional Ethical Committee. Written consent for the use of patient data was obtained from all participants.

Tumor tissues were obtained from each patient via surgery or biopsy and formalin-fixed paraffin-embedded (FFPE). Owing to the availability of sufficient biopsy material for whole-exome sequencing and PD-L1 assessment, PD-L1 immunohistochemistry (IHC) was performed on archival tumor specimens after completion of the initial diagnostic IHC evaluations and genomic sequencing procedures. PD-L1 expression was evaluated in tumor samples by immunohistochemistry with the PD-L1 Dako22C3 pharmDx antibody (Agilent Technologies; Carpinteria, CA, USA). The tumor proportion score (TPS) was determined as the percentage of PD-L1-positive tumor cells, with TPS ≥ 1% defined as positive. The combined proportion score (CPS) was calculated as the percentage of PD-L1-positive cells (including tumor cells, lymphocytes, and macrophages) divided by the total number of viable tumor cells, with CPS ≥ 1% also considered positive [21]. Genomic alterations were assessed using next-generation sequencing (NGS). Briefly, genomic DNA was extracted from paraffin-embedded biopsy tissue for each patient, followed by preparation of sequencing libraries and whole-exome sequencing according to the manufacturer’s protocols. Raw FASTQ files underwent quality control using Trimmomatic (v0.39, Aachen, German). Paired-end reads were aligned to the human reference genome (hg19) using BWA-MEM (v0.7.17; Broad Institute, Cambridge, MA, USA). Somatic mutations, including single nucleotide variants (SNVs) and insertions/deletions (indels), were identified and annotated with ANNOVAR (2020-06-08 release, Philadelphia, PA, USA), with manual review performed using the Integrative Genomics Viewer (IGV, v2.9.4, Broad Institute, Cambridge, MA, USA). Copy number variations (CNVs) were identified using CNVnator (v0.4.1, Princeton, NJ, USA). Additionally, microsatellite instability (MSI) status was evaluated by analyzing mutations in 15 MSI-related genes, with MSI-high (MSI-H) defined as a mutation rate of ≥30% [22].

Tumor response was evaluated every 8 weeks using CT scans, or MRI as needed. Independent radiologic review assessed treatment efficacy according to the Response Evaluation Criteria in Solid Tumors (RECIST) version 1.1, focusing on changes in tumor size. Adverse events were monitored and graded using the Common Terminology Criteria for Adverse Events (CTCAE, version 4.0). The primary objective was progression-free survival (PFS), defined as the time from treatment initiation to disease progression or death from any cause, based on RECIST 1.1 criteria. Secondary endpoints included the following: objective response rate (ORR), defined as the proportion of patients achieving complete or partial response; disease control rate (DCR), encompassing complete response, partial response, or stable disease; overall survival (OS), measured from treatment start to death from any cause. The efficacy-evaluable population comprised patients who received at least one cycle of study treatment and underwent at least one post-baseline radiologic assessment according to RECIST version 1.1. Patients with missing post-baseline imaging assessments, owing to early discontinuation, loss to follow-up, or other causes, were excluded from the primary analyses of ORR, DCR, and PFS in a conservative approach to minimize bias. All enrolled patients were included in the intent-to-treat (ITT) population for safety analyses. An exploratory analysis was conducted to assess the predictive value of PD-L1 expression and genomic alterations on clinical outcomes.

### 2.4. Statistical Analyses

Clinicopathological characteristics, treatment efficacy, and adverse events (AEs) were summarized using descriptive statistics. Progression-free survival (PFS) and overall survival (OS) were estimated using the Kaplan–Meier method, with medians and 95% confidence intervals (CIs) reported. Comparisons of PFS between subgroups were performed using log-rank tests, with a two-sided *p*-value < 0.05 considered statistically significant. PFS was defined as the duration from the start of treatment until disease progression or the last follow-up, and OS was defined as the time from treatment initiation to death from any cause, with censoring at the last follow-up for surviving patients. All statistical analyses were conducted using IBM SPSS version 20.0 (IBM Corp., Armonk, NY, USA).

Because a proportion of patients did not have evaluable PD-L1 results, analyses were conducted using both complete-case methods and multiple imputation (MI) to ensure robustness. For the primary analyses, only patients with complete information on PD-L1 status, survival time, and event status were included (complete-case dataset). Cox proportional hazards models were used to evaluate the association between PD-L1 expression (positive vs. Negative) and PFS or OS. To evaluate the potential influence of missing PD-L1 data, multiple imputation (MI) was performed using a chained-equations framework. The imputation model included PFS, PFS event status, OS, OS event status, and PD-L1 status. Five imputed datasets (m = 5) were generated using iterative regression with posterior sampling. In each imputed dataset, PD-L1 values were rounded to binary categories (positive vs. Negative) and Cox models were fitted separately for PFS and OS. Log hazard ratios and variances from the five models were pooled using Rubin’s rules to obtain the MI-combined HRs and 95% CIs.

A null hypothesis of a 6-month progression-free survival (PFS) rate of 30% was tested against an alternative hypothesis of a 50% PFS rate using a one-sided exact binomial test at an α level of 2.5%. Enrolling 30 patients provides 80% power to detect 20% absolute improvement, justified by the need to balance adequate statistical power with the feasibility of recruiting patients for a rare cancer like cholangiocarcinoma, where large sample sizes are challenging to achieve.

## 3. Results

### 3.1. Patient Characteristics

Between April 2019 and October 2021, a total of 30 patients with advanced BTC were enrolled in the study. The baseline clinicopathological characteristics of the patients are summarized in Table 1. The median age was 62 years (range 44–73), 66.7% were aged over 60 years and 43.3% of patients were female. Eastern Cooperative Oncology Group (ECOG) performance status scores were one in twenty patients (66.7%) and two in ten patients (33.3%). The majority of patients had intrahepatic cholangiocarcinoma (n = 22; 73.3%), followed by extrahepatic cholangiocarcinoma (n = 5; 16.7%) and gallbladder cancer (n = 3; 10.0%). Most patients (n = 26; 86.7%) presented with metastatic disease, with the most common sites being lymph nodes (n = 21; 70.0%) and lungs (n = 9; 30.0%). A history of hepatitis B surface antigen (HBsAg) positivity was noted in 12 (40%) of patients, all of whom had received antiviral therapy during antitumor treatment. Baseline evaluations revealed elevated lactate dehydrogenase (LDH) levels in 11 patients (36.7%), carbohydrate antigen 199 (CA19-9) in 21 patients (70.0%), and carcinoembryonic antigen (CEA) in 11 patients (36.7%).

### 3.2. Treatment and Efficacy

The median follow-up duration was 16.0 months. While all patients received at least one treatment cycle, 23 fulfilled these requirements and were included in the efficacy analyses. The other seven patients were considered non-evaluable owing to early dropout (n = 4) related to treatment-associated adverse effects or disease progression, including fever (one case), malaise (two cases), and hematemesis (one case), or loss to follow-up without documented cause (n = 3; among these, one patient completed one cycle and two received three cycles). Overall, patients completed a median of two and a half cycles of chemotherapy plus immunotherapy (range, one to four) and five cycles of immunotherapy maintenance (range, one to eighteen). None of the participants completed immunotherapy maintenance cycles. Among responders, patients achieving partial response (PR) underwent an average of 6.3 cycles: those with stable disease (SD) received 6.9 cycles, and those with progressive disease (PD) received 3.25 cycles.

**Figure 1:**
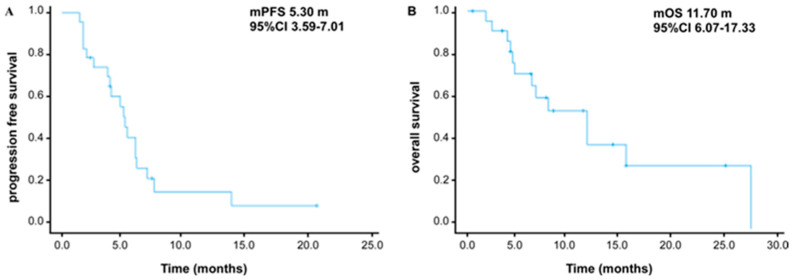
Therapeutic efficacy of toripalimab in combination with gemcitabine and 5-fluorouracil in patients with advanced or metastatic biliary tract carcinoma. (**A**) Kaplan-Meier estimation of progression-free survival of 23 patients with complete radiological response evaluation. (**B**) Kaplan-Meier estimation of overall survival of the entire cohort.

The treatment efficacy outcomes are summarized in Table 2. In accordance with RECIST v1.1 criteria, among the 23 patients evaluable for efficacy, no complete responses (CRs) were observed. Three patients (13.0%) achieved a partial response (PR), with response durations ranging from 6.3 to 21.8 months. Sixteen patients (69.6%) demonstrated stable disease (SD), whereas four patients (17.4%) exhibited progressive disease (PD). Although the observed objective response rate (ORR) was 13.0%, the statistical analysis confirmed that this did not meet the prespecified primary endpoint of ORR ≥30%. Nonetheless, the disease control rate (DCR) of 82.6% (19/23) reflects the predominance of PR and SD outcomes. For the entire cohort, the median PFS was 5.3 months (95% CI: 3.6–7.01), and the median OS was 11.7 months (95% CI: 6.1–17.3) (Figure 1A,B).

These survival outcomes were consistent with the clinical activity suggested by the high DCR, indicating durable disease stabilization despite the modest ORR. Changes in serum CA-199 and CEA levels over the treatment course suggested that elevated tumor markers may be associated with a trend toward increased tumor burden and fewer treatment cycles received (Figure S1A,B). Univariate Cox regression analysis was performed to identify potential prognostic factors associated with survival outcomes (Table 3). Among the evaluated clinical and biomarker variables, ECOG performance status was the only parameter significantly associated with poorer survival (HR = 3.07, 95% CI: 1.13–8.30; *p* = 0.027). Age demonstrated a borderline association with increased risk (HR = 2.61, 95% CI: 0.98–6.96; *p* = 0.056), and elevated CEA levels showed a similar trend (HR = 2.33, 95% CI: 0.88–6.13; *p* = 0.088), although neither reached statistical significance. Other variables, including gender, TMB, PD-L1 CPS, LDH, CA19-9, and HBsAg status, did not exhibit significant associations with survival. These findings indicate that baseline functional status, rather than tumor-related biomarkers, serves as the primary prognostic indicator within this cohort. Due to the limited number of survival events, multivariate Cox regression could not be reliably performed, and therefore only univariate results are reported.

**Figure 2:**
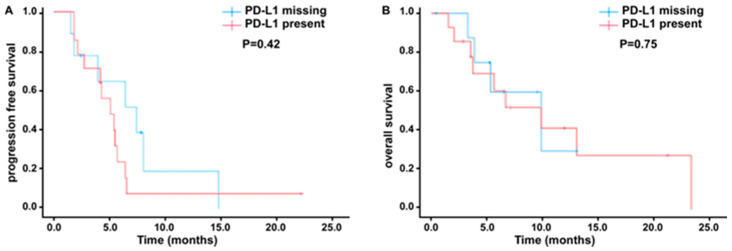
Kaplan–Meier survival analysis according to PD-L1 status. (**A**) Progression-free survival (PFS) in patients stratified by PD-L1 expression (PD-L1 present vs. PD-L1 missing). (**B**) Overall survival (OS) in the same cohort stratified by PD-L1 expression status.

**Figure 3:**
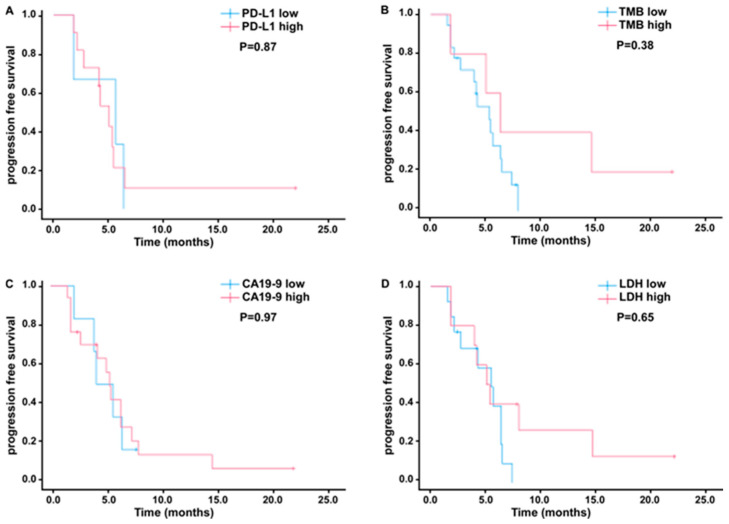
Subgroup analyses of progression-free survival (PFS). (**A**) Kaplan-Meier plot for PFS based on the expression of PD-L1-positive (N = 15) and PD-L1-negative (N = 4) (combined proportion score). (**B**) Kaplan-Meier plot for PFS for patients with high TMB (≥6 Muts/Mb) or low TMB (<6 Muts/Mb). (**C**) Kaplan-Meier plot for PFS for patients with high CA19-9 (≥37 U/mL) or low CA19-9 (<37 U/mL). (**D**) Kaplan-Meier plot for PFS for patients with high LDH (≥250 U/L) or low LDH (<250 U/L).

Exploratory analyses were conducted to evaluate the associations between PD-L1 expression, tissue tumor mutation burden (TMB), and mismatch repair (MMR) gene status to clinical responses of toripalimab in combination with gemcitabine and 5-fluorouracil (5-FU). Among 30 patients, PD-L1 status was available for 19 (63.3%) and missing for 11 (36.7%); reasons for missingness are insufficient tissue. Eight patient (42.1%) exhibited positive tumor PD-L1 expression (TPS > 1%) and 15 (78.9%) showed positive PD-L1 expression (CPS ≥ 1%). Prognostic factors including age, sex, tumor stage, histologic subtype, CA19-9, CEA, and LDH were comparable between patients with available and those with missing PD-L1 results, with all tests yielding *p*-values > 0.05 (Table 4). The objective response rate (ORR) was similar in the overall cohort (63.3%) and in the PD-L1-positive subgroup (66.7%). Descriptive survival analyses demonstrated similar crude event rates and overlapping outcomes by PD-L1 testing availability (Table 5). Kaplan–Meier curves stratified by PD-L1 availability showed that median PFS was 4.1 months in the PD-L1-available group versus 1.8 months in the missing group (log-rank *p* = 0.30), and median OS was 7.7 versus 6.3 months, respectively (log-rank *p* = 0.98) (Figure 2). To further evaluate the potential influence of missing PD-L1 values, we conducted sensitivity analyses using multiple imputation (MI) via a chained-equations framework. Five imputed datasets (*m* = 5) were generated incorporating PFS, OS, event status, and PD-L1 status. Cox models were fitted within each dataset, and the estimates were pooled using Rubin’s rules. The MI-combined results remained fully consistent with the complete-case findings: PFS (MI-pooled) (HR = 0.90, 95% CI 0.26–3.05, *p* = 0.86) and OS (MI-pooled) (HR = 1.00, 95% CI 0.24–4.15, *p* = 1.00), respectively. Collectively, these results indicate the bias that inference of PD-L1 is not a significant prognostic marker in this cohort. The median TMB in our cohort was 4.6 muts/Mb (range, 0–16.1), higher than the previously reported median of 1.25 muts/Mb (range, 0.03–54.74) [23]. This difference likely reflects patient heterogeneity and small sample size; additionally, the prior study included only intrahepatic cholangiocarcinoma, which may account for the lower TMB observed. No statistically significant association was observed between PD-L1 expression (*p* = 0.87) or TMB (*p* = 0.44) and PFS, a finding that may be partially attributable to limited cohort size and variability in clinical response (Figure 3A,B). Consistently, Kaplan–Meier survival analyses demonstrated that neither pretreatment CA19-9 levels (*p* = 0.97) nor LDH levels (*p* = 0.65) were predictive of PFS (Figure 3C,D). Moreover, stratification by age, gender, CEA levels, and HBV infection status also revealed no significant differences in PFS, indicating that these baseline clinical and biomarker characteristics did not confer measurable prognostic value in this study population. (Figure S2).

### 3.3. Mutational Profile of Tumors

**Figure 4:**
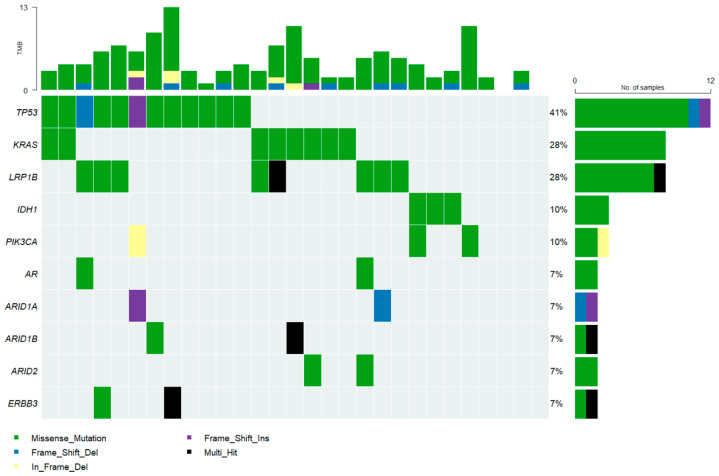
Landscape of somatic mutations and tumor mutational burden (TMB) across the cohort. Waterfall plot summarizing the distribution of tumor mutational burden (**top panel**) and the frequency and types of somatic alterations detected in the analyzed samples (**central panel**). Each column represents an individual tumor sample, and each row corresponds to one of the most frequently mutated genes. Mutation types are indicated by color: missense mutation (green), frame-shift deletion (blue), in-frame deletion (yellow), frame-shift insertion (purple), and multi-hit events (black). The bar plot on the **right** displays the percentage of samples harboring mutations in each gene. TMB values are shown as bars above each sample and expressed as mutations per megabase (mut/Mb).

Next-generation sequencing performed on 29 tissue samples detected the most frequent mutations in TP53 (41.0%), KRAS (28%), FGFR1/2 (28%), LRP1B (28%), IDH1 (10%), PIK3CA (10%), AR (7%), ARID1A (7%), ARID1B (7%), and ERBB3 (7%) (Figure 4). DNA damage response (DDR)-related gene mutations are being explored in BTC as actionable biomarkers for systemic therapies, including platinum-based chemotherapy and PARP inhibitors [24]. In our study, DDR-related mutations occurred in 72% (21/29) of cases, affecting mismatch repair (MMR), homologous recombination repair (HRR), nucleotide excision repair (NER), non-homologous end joining (NHEJ), and base excision repair (BER) (Figure S3).

### 3.4. Second-Line Treatment Regimens

Upon disease progression on the study regimen, patients were allowed to receive subsequent systemic or regional therapies, including mFOLFOX6 chemotherapy, FGFR-targeted therapy, anti-HER2 therapy, and regional therapies. Rebiopsy was performed in two patients upon progression to explore potential resistance-related genomic alterations. One patient harboring an FGFR2 mutation progressed during toripalimab maintenance and subsequently received the FGFR inhibitor ICP-192 and palliative radiotherapy to lumbar spine metastasis, followed by nab-paclitaxel plus cisplatin upon further progression. Another patient with ERBB2 amplification progressed on with toripalimab maintenance and was treated with trastuzumab, pertuzumab, and cisplatin for four cycles, followed by maintenance with trastuzumab and pertuzumab. This patient later derived clinical benefit from lenvatinib for an additional seven months before transitioning to supportive care, achieving an overall survival of approximately 30 months.

### 3.5. Safety

All patients (100%) experienced at least one treatment-related adverse event (TRAE), and eight patients (26.7%) developed grade ≥ 3 TRAEs (Table 6). The most common all-grade toxicities were fatigue (89.9%) and nausea (86.7%). Grade ≥3 TRAEs included anemia (20.0%), leukopenia (13.3%), and thrombocytopenia (10.0%). Immune checkpoint inhibitor-related adverse events primarily consisted of hypothyroidism (10.0%), hypocortisolemia (6.6%), pneumonitis (3.3%), and hyperthyroidism (3.3%). All TRAEs were manageable with standard supportive care. No severe immune-mediated toxicities or treatment-related deaths occurred.

## 4. Discussion

Immunotherapy-based regimens, particularly immune checkpoint inhibitors combined with gemcitabine and cisplatin, have emerged as the standard first-line treatment for advanced biliary tract cancer (BTC), offering meaningful improvements in overall survival compared with chemotherapy alone. Nevertheless, treatment options remain limited, and the development of novel immunotherapy combinations remains an important clinical priority. In this study, toripalimab combined with the GCF regimen demonstrated encouraging antitumor activity in patients with advanced or metastatic BTC, yielding an ORR of 10% and a median OS of 7.7 months. Grade 3 or higher treatment-related adverse events were observed in 26.7% of subjects, consistent with the expected safety profile of chemo-immunotherapy combinations.

Before anti-PD-1/PD-L1, antibodies were increasingly used in both first- and second-line treatment of BTC, Nivolumab plus chemotherapy yielded an ORR of 33.3% and superior outcomes compared with monotherapy [25]. In our study, the ORR was 13.0%, likely reflecting the fact that none of the patients completed maintenance immunotherapy. This result is consistent with the retrospective findings of Sun et al., who reported an ORR of 13% and DCR of 34% for PD-1 inhibitors combined with chemotherapy [26]. Pembrolizumab demonstrated modest activity (ORR 5.8%), with only marginal benefit in PD-L1-positive patients [27]. The lack of ORR difference by PD-L1 status in our study may be attributable to the small number of PD-L1-positive cases. Conversely, camrelizumab-based combinations have reported higher activity, with ORRs up to 54% and DCRs of 73.3% in advanced intrahepatic BTC [28,29]. Differences between intrahepatic and extrahepatic disease, tumor immune microenvironment, and variable sensitivity to PD-1 inhibitors and gemcitabine-based regimens may all contribute to the divergent efficacy observed across studies. A summary of representative immunotherapy combination strategies is provided in Table 7.

Intrahepatic BTC is frequently characterized by immunologically “cold” phenotypes, which are associated with limited responsiveness to PD-1 inhibitor monotherapy [25]. Samuel et al. similarly reported significantly lower immunotherapy efficacy in patients with advanced stage tumors [30]. Consistent with these observations, 73.3% of patients in our study had intrahepatic BTC and 86% had stage IV disease, which may partly contribute to the modest ORR observed. Multiple combination regimens are currently under investigation to improve outcomes in this population. Shi et al. reported an ORR of 80% for toripalimab combined with Gemox and lenvatinib, which was notably higher than that achieved in our cohort [31]. Patients with lower tumor burden may be more likely to tolerate and benefit from such intensified therapies, although careful patient selection is required. Additional studies evaluating combinations such as durvalumab plus tremelimumab with chemotherapy and the antiangiogenic agent bevacizumab are ongoing and are expected to further inform treatment strategies for BTC [32].

Our study also demonstrated that tissue TMB was not associated with clinical response to toripalimab combined with GCF. This finding aligns with report by Frega et al., which showed no significant correlation between TMB and immunotherapy outcomes in BTC [33]. Similarly, PD-L1 expression on tumor cells or tumor-infiltrating immune cells did not predict treatment response in our cohort. Although some studies have suggested that tumor-cell PD-L1 expression may correlate with benefit as compared to immune-cell PD-L1, the majority of available data indicate no meaningful differences in PD-L1 expression across anatomical BTC subtypes [34]. Moreover, BTC is generally considered as an immune desert or “cold” malignancy with inherently low immunogenicity, as highlighted in both the TOPAZ-1 and KEYNOTE-966 trials, which demonstrated only modest responsiveness to immunotherapy [35,36]. However, these findings must be interpreted cautiously due to the small sample size and limited number of events. Larger studies are needed to clarify the predictive value of TMB and PD-L1 expression, particularly in the context of PD-1/PD-L1 inhibitors combined with chemotherapy. Further investigations should continue to explore reliable biomarkers and refine patient selection for immunotherapy-based regimens in BTC.

**Table 7:** Key clinical trials of PD-1/PD-L1 inhibitors combined with chemotherapy as first-line treatment for advanced biliary tract cancer.

Study (Lead Author et al., Year)	Clinical Trial No.	Study Design	Agents	Study Population (n)	Patients	Clinical Stage (%)	mPFS (Months)	mOS (Months)	DCR (%)
TOPAZ-1 (Oh et al., 2022; updated 2024) [35]	NCT03875235	Phase 3, randomized, double-blind, placebo-controlled	Durvalumab + GemCis vs. Placebo + GemCis	341 vs. 344 (total 685)	First-line unresectable/metastatic BTC	Locally advanced: 44%; Metastatic: 56%	7.2 vs. 5.7	12.9 vs. 11.3	80.4 vs. 78.0
KEYNOTE-966 (Kelley et al., 2023) [12]	NCT04003636	Phase 3, randomized, double-blind, placebo-controlled	Pembrolizumab + GemCis vs. Placebo + GemCis	533 vs. 536 (total 1069)	First-line unresectable/metastatic BTC	Locally advanced: 12%; Metastatic: 88%	6.5 vs. 5.6	12.7 vs. 10.9	82.6 vs. 75.3
Shi et al., 2023 [31]	NCT03951597	Phase 2, single-arm, open-label	Toripalimab + lenvatinib + GEMOX	30	First-line advanced intrahepatic cholangiocarcinoma	Locally advanced: 0%; Metastatic: 100%	10.0	22.5	93.3
Shi et al., 2025 (AdvanTIG-105/ZSAB-TOP) [37]	NCT05023109	Phase 2, open-label, multicentre	Tislelizumab + ociperlimab + GemCis	41	First-line unresectable advanced BTC	Locally advanced: ~15%; Metastatic: ~85%	9.7	19.3	82.9
Chen/Xie et al., 2020–2023 [28]	NCT03486678	Phase 2, single-arm, open-label	Camrelizumab + GEMOX	38–54	First-line advanced BTC	Locally advanced: ~30%; Metastatic: ~70%	7.0	14.9	86.8–91
Li et al., 2022 [38]	NCT03796429	Phase 2, single-arm, open-label	Toripalimab + gemcitabine + S-1	50	First-line advanced BTC	Locally advanced: ~20%; Metastatic: ~80%	7.0	15.0	87.8
Ueno et al., 2019–2022 (NivoGem Japanese study) [25]	JapicCTI-153098	Phase 1/2, single-arm	Nivolumab + GemCis	30	First-line unresectable/recurrent BTC	Locally advanced: ~25%; Metastatic/recurrent: ~75%	5.2	15.4	90.0
IMbrave151 (Macarulla et al., 2024) [39]	NCT04677504	Phase 2, randomized, double-blind, placebo-controlled	Atezolizumab + bevacizumab + GemCis vs. Atezolizumab + placebo + GemCis	79 vs. 83	First-line advanced BTC	Locally advanced: 18%; Metastatic: 82%	8.4 vs. 7.9	14.9 vs. 14.6	~85 vs. ~80

Abbreviations: BTC = biliary tract cancer; GemCis = gemcitabine + cisplatin; GEMOX = gemcitabine + oxaliplatin; mPFS = median progression-free survival; mOS = median overall survival; DCR = disease control rate (CR + PR + SD).

The most frequently mutated genes identified in our cohort—TP53, KRAS, FGFR, IDH1, PIK3CA, and BRCA1/2—are consistent with previously reported mutation rates of approximately 2–29% in BTC [10,40]. Notably, 72% of patients in our cohort harbored mutations in DDR-related gene. Defects in DDR pathways can promote genomic instability, increase TMB and neoantigen load, and enhance tumor immunogenicity, theoretically rendering tumors more susceptible to immune checkpoint inhibition, including PD-1/PD-L1 blockade such as toripalimab [24,41,42]. However, our study observed only a moderate response to ICIs, lower than expected given the presumed immunogenic potential of DDR alterations [42]. This discrepancy highlights the need for further investigation into factors such as temporal heterogeneity in DDR mutations, variability in neoantigen presentation, and the complex tumor microenvironment of BTC. Despite these limitations, DDR deficiency remains a promising therapeutic target, and ongoing genomic and clinical studies—including trials evaluating PARP inhibitors in combination with ICIs—may better define its clinical relevance in BTC. In our study, several patients received targeted therapies based on actionable alterations identified through NGS and achieved relatively favorable outcomes, underscoring the importance of NGS in guiding individualized treatment strategies. Targeted therapies currently under investigation include FGFR1/2/3 inhibitors such as pemigatinib and infigratinib, as well as VEGF-directed agents including monoclonal antibodies and multireceptor tyrosine kinase inhibitors [43,44,45].

In this study, the most common adverse events were elevations in liver enzymes and hematologic toxicities, consistent with previously reported safety profiles of chemotherapy and anti-PD-1 antibodies in biliary tract carcinoma. Grade 3 and over-adverse events occurred in 26.7% of patients and were manageable and reversible with appropriate supportive care. By comparison, a phase II trial evaluating toripalimab combined with Gemox and lenvatinib as first-line therapy for advanced intrahepatic BTC reported grade ≥3 adverse events in 56.7% of patients, suggesting that more intensive regimens may be less suitable for patients with advanced disease [33]. No treatment-related deaths were observed in our study. Approximately half of the enrolled patients were HBsAg positive; however, hepatitis reactivation was not observed among individuals receiving antiviral prophylaxis, underscoring the feasibility of immunotherapy-based regimens in this subset. Overall, these findings indicate that the combination regimen demonstrates an acceptable safety profile for patients with advanced or metastatic BTC.

This study provides a preliminary evaluation of the efficacy and safety of toripalimab combined with gemcitabine and 5-FU for advanced BTC, demonstrating a manageable toxicity profile. However, several limitations of this single-arm phase II trial should be considered when interpreting the findings. First, the small sample size restricts statistical power and limits the generalizability of the findings. PD-L1 expression was evaluable in only 63.3% of patients, and tumor response could not be assessed in 23.3% of the cohort, further constraining the robustness of biomarker and efficacy analyses. In addition, the majority of enrolled patients had metastatic disease with substantial tumor burden, which may partly explain the lower response rates compared with those typically observed in patients with locally advanced disease. Many patients with high tumor burden were also unable to complete the planned treatment cycles and subsequently received additional systemic therapies or local therapies after progression. Local treatments, including radiofrequency ablation, TACE, and radiotherapy, were used to manage metastatic lesions, potentially confounding the interpretation of overall survival outcomes [46,47]. These limitations highlight the need for future investigations to explore PD-1 inhibitors combined with chemotherapy in conjunction with local treatments, particularly in patients with locally advanced BTC who may derive greater benefit from multimodal therapeutic strategies.

## 5. Conclusions

The combination of toripalimab with gemcitabine and 5-fluorouracil showed acceptable safety and potential efficacy for patients with advanced or metastatic biliary tract carcinoma, particularly in subgroups ineligible for intensive platinum-based therapies.

## Figures and Tables

**Table 1:** Patient clinical pathological characteristics.

Characteristics	n (%)
Sex	
Male	17 (56.6%)
Female	13 (43.3%)
Age	
<60	10 (33.3%)
≥60	20 (66.7%)
ECOG performance status	
0~1	20 (66.7%)
2	10 (33.3%)
Primary tumor location	
Intrahepatic	22 (73.3%)
Extrahepatic	5 (16.7%)
Gallbladder	3 (10.0%)
PD-L1 expression (CPS)	
<1%	4 (13.3%)
>1%	15 (50.0%)
unknown	11 (36.7%)
Clinical stage	
II-IIIB	4 (13.3%)
IV	26 (86.7%)
Site of metastasis	
Lymph nodes	21 (70.0%)
Liver	1 (3.30%)
Lung	9 (30.0%)
Bone	3 (10.0%)
Others	3 (10.0%)
HBsAg	
Negative	18 (60.0%)
Positive	12 (40.0%)
LDH (U/L)	
<250	19 (63.3%)
>250	11 (36.7%)
CA 199 (U/mL)	
<37	8 (26.7%)
>37	22 (73.3%)

Abbreviations: ECOG, Eastern Cooperative Oncology Group; CPS, combined proportion score; LDH, lactate dehydrogenase; CA19-9, carbohydrate antigen 19-9.

**Table 2:** Best overall response.

	(n/%)
Complete response	0 (0%)
Partial response	3 (13.0%)
Stable disease	16 (69.6%)
Progressive disease	4 (17.4%)
No measurable disease	7

**Table 3:** Univariate Cox regression analysis of clinical and biomarker variables for PFS.

Variable	HR (Exp(B))	95% CI	*p*-Value
Age	2.605	0.975–6.961	0.056
Gender	0.972	0.372–2.535	0.953
ECOG PS	3.067	1.134–8.295	0.027
TMB	0.429	0.121–1.527	0.191
PD-L1 CPS	1.039	0.267–4.046	0.956
LDH	0.547	0.198–1.513	0.245
CEA	2.326	0.883–6.132	0.088
CA19-9	0.913	0.321–2.601	0.865
HBsAg	1.279	0.498–3.285	0.609

**Table 4:** Baseline clinicopathologic characteristics by PD-L1 testing availability (present vs. missing).

Variable	PD-L1 Present (n = 19)	PD-L1 Missing (n = 11)	*p*-Value
Age, median (y)	64	61	0.31
Sex (male)	11	6	1.00
Stage IV (%)	79%	82%	0.84
HBsAg positive	9	3	0.44
ECOG, median	1	1	0.31
CA19-9, median (U/mL)	13.96	2.51	0.41
CEA, median (ng/mL)	3.11	1.79	0.85
LDH, median (U/L)	210	220	0.76
TMB, median (Muts/Mb)	4.6	4.6	0.73

**Table 5:** Descriptive summaries of PFS and OS by PD-L1 testing availability (present vs. missing).

Endpoint	PD-L1 Testing Availability	n	Events	Censored	Median (95% CI), m	6 m Survival %	12 m Survival %	Log-Rank *p*-Value
PFS	Present	14	12	2	5.0 (3.2–11.8)	8.0	8.0	0.42
PFS	Missing	9	7	2	7.3 (2.9–11.8)	52.0	22.0	
OS	Present	14	9	6	11.7 (4.1–19.3)	61.0	42.0	0.75
OS	Missing	9	4	5	11.7 (3.6–19.8)	61.0	37.0	

**Table 6:** Treatment-related adverse events.

	Grades 1~2 n (%)	Grades ≥ 3 n (%)
Overall	30	8
Leukocytopenia	17 (56.6)	4 (13.3)
Anemia	17 (56.6)	6 (20.0)
Platelet count decreased	13 (43.3)	3 (10)
Nausea	27 (89.9)	2 (6.6)
Vomiting	19 (63.3)	2 (6.6)
Constipation	14 (46.6)	0 (0)
Diarrhea	3 (10)	1 (3.3)
Febrile	8 (26.6)	3 (10)
Rash	7 (23.3)	1 (3.3)
Hypothyroidism	3 (10)	0 (0)
Hyperthyroidism	1 (3.3)	0
Fatigue	26 (86.7)	1 (3.3)
Alanine aminotransferase increased	5 (16.7)	0 (0)
Aspartate aminotransferase increased	7 (23.3)	1 (3.3)
Blood bilirubin increased	3 (10)	1 (3.3)
Sepsis	0 (0)	2 (6.6)
Upper gastrointestinal hemorrhage	1 (3.3)	1 (3.3)
Intracranial bleed	0 (0)	1(3.3)
Adrenocortical insufficiency	2(6.6)	1(3.3)
Pneumonitis	1(3.3)	0 (0)

## Data Availability

The data presented in this study are available on request from the corresponding author.

## Supplementary Materials

The following supporting information can be downloaded at https://www.mdpi.com/article/10.3390/cancers18010088/s1; Figure S1 Serum CA19-9 and CEA levels in patients receiving combined therapy. Concentrations of CA19-9 (A) and CEA (B) at baseline and each treatment course in the cohort. Figure S2 Subgroup analyses of progression-free survival (PFS). (A,B) Kaplan–Meier plot showed significant difference of PFS for patients between high and low serum CA19-9 or LDH levels, respectively. (C,D) The Kaplan-Meier analysis showed no significant difference in progression-free survival (PFS) between patients with high and low serum CEA levels or between HBsAg-positive and HBsAg-negative patients. Figure S3 Top 10 genomic alterations of genes in patients advanced biliary tract carcinoma receiving combined therapy.

## Abbreviations

The following abbreviations are used in this manuscript:

biliary tract carcinoma	BTC
tumor mutational burden	TMB
lactate dehydrogenase	LDH
progression-free survival	PFS
overall survival	OS
objective response rate	ORR
disease control rate	DCR
tumor proportion score	TPS
combined proportion score	CPS
microsatellite instability	MSI
Transarterial chemoembolization	TACE
Carbohydrate antigen199	CA19-9
carcinoembryonic antigen	CEA
