# Supplementary figures and images for: A Phase II Study of Toripalimab in Combination with Gemcitabine and 5-Fluorouracil as First-Line Therapy for Advanced or Metastatic Biliary Tract Carcinoma
Source: Cancers (Basel). 2025 Dec 27;18(1):88. doi: 10.3390/cancers18010088 (PMC12784805; doi:10.3390/cancers18010088)

**A**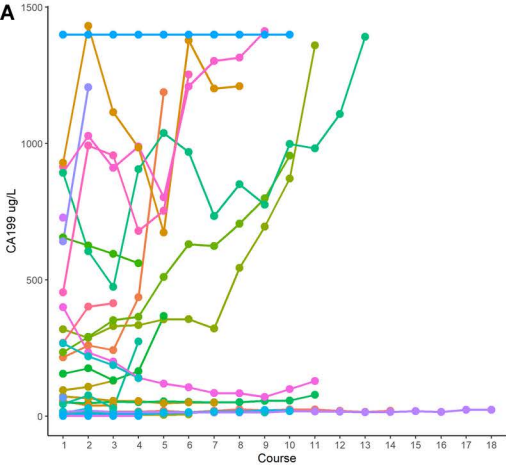**B**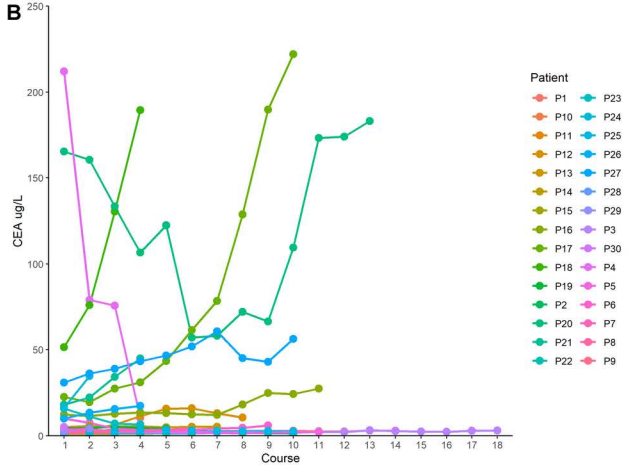

Supplement: Supplementary file 1 [file cancers-18-00088-s001.zip › Supplemental Figure S1.pdf]

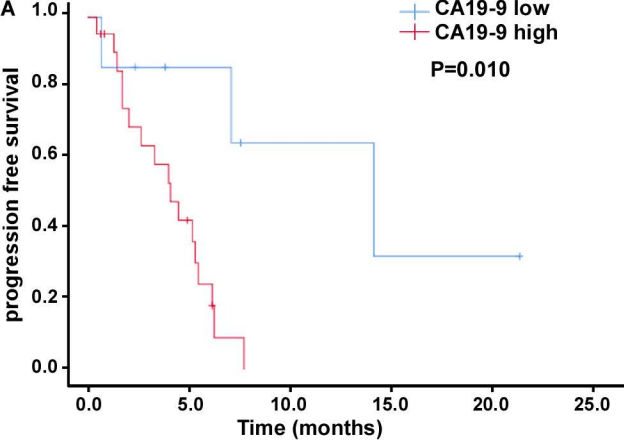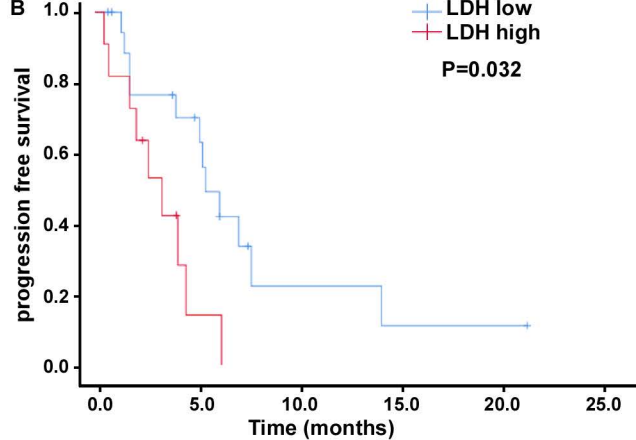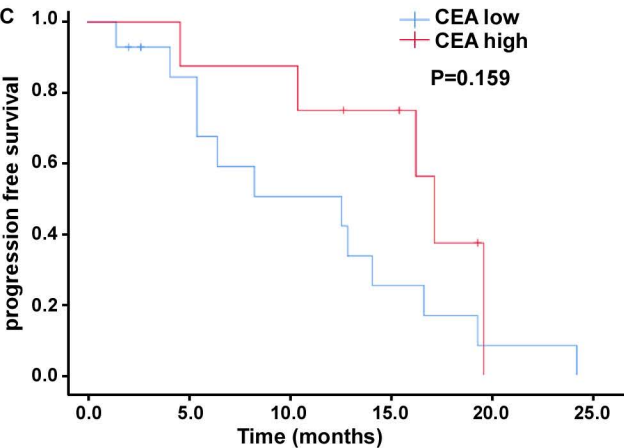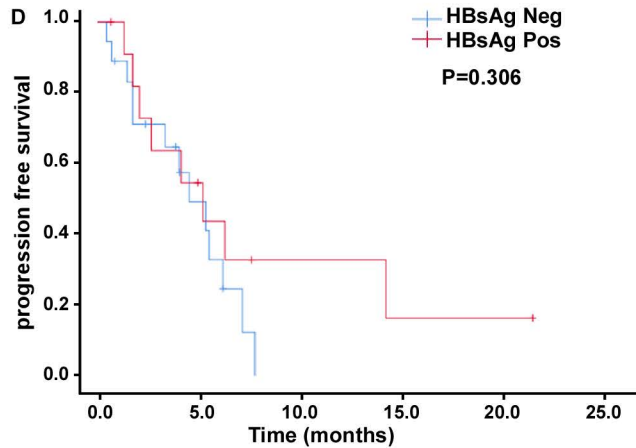

Supplement: Supplementary file 1 [file cancers-18-00088-s001.zip › Supplemental Figure S2.pdf]

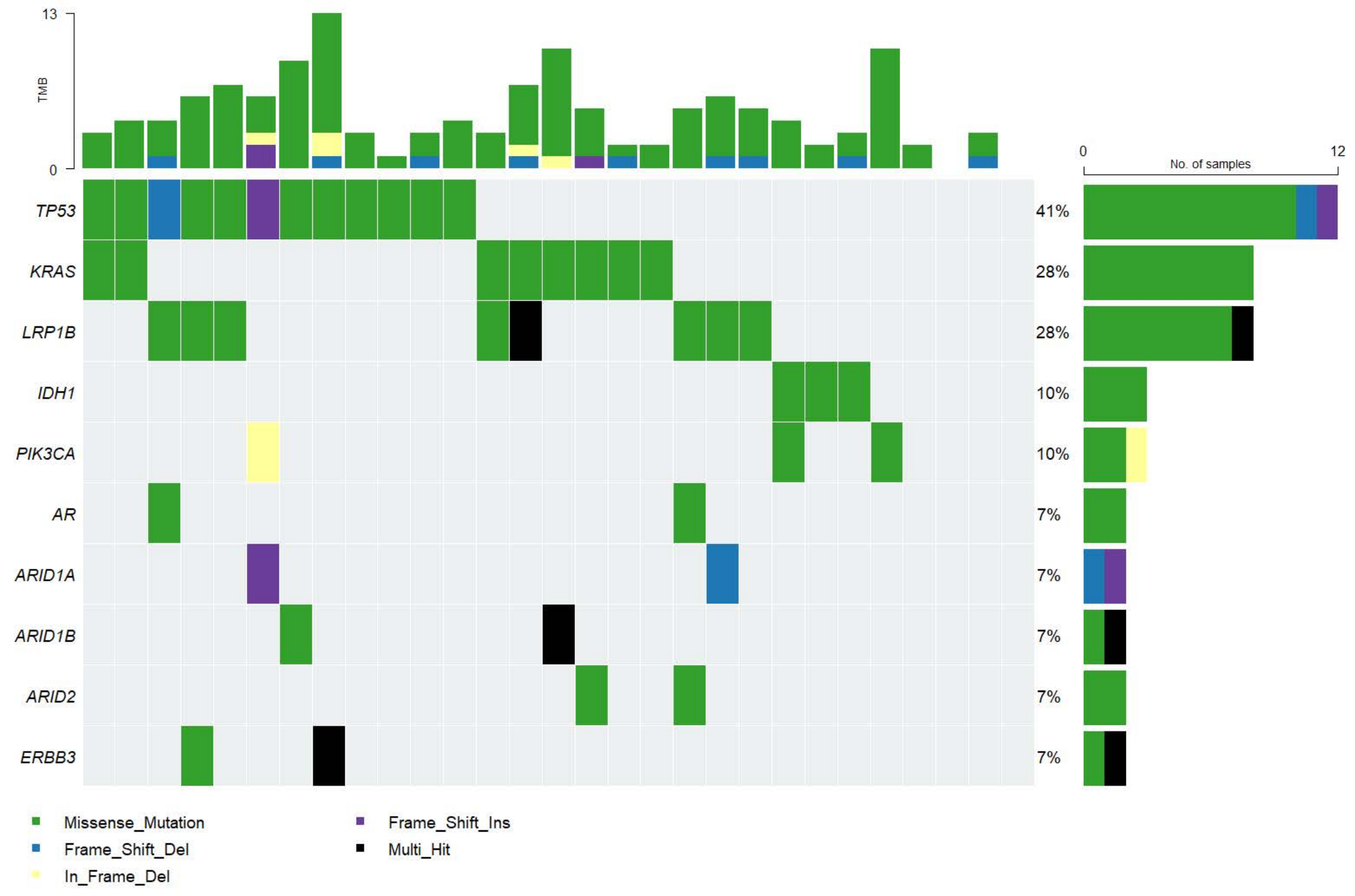

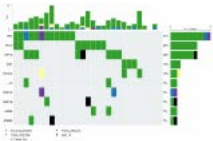

Supplement: Supplementary file 1 [file cancers-18-00088-s001.zip › Supplemental Figure S3.pdf]
